# Prevalence and factors associated with occupational hazard exposure among undergraduate veterinary students in Bangladesh

**DOI:** 10.1016/j.pmedr.2025.103196

**Published:** 2025-08-07

**Authors:** Pronab Das, Seemanta Das, Muhammad Abdul Mannan, Sharmin Chowdhury

**Affiliations:** a One Health Institute, Chattogram Veterinary and Animal Sciences University, Chattogram 4225, Bangladesh; b Department of Microbiology and Parasitology, Sher-e-Bangla Agricultural University, Dhaka 1207, Bangladesh; c Department of Pathology and Parasitology, Chattogram Veterinary and Animal Sciences University, Chattogram 4225, Bangladesh

**Keywords:** Occupational hazard, Occupational health, Veterinary student, Prevalence, Factor, Bangladesh

## Abstract

**Objectives:**

Veterinarians are observed to be vulnerable to various occupational hazards due to the nature of their work. Veterinary students face an even greater risk, as their academic and professional training in animal handling is still incomplete. Therefore, this study aimed to investigate the prevalence of and factors associated with occupational hazard exposure among undergraduate veterinary students in Bangladesh.

**Methods:**

A cross-sectional study was conducted from August 25, 2024 to September 10, 2024 among 330 veterinary students from two conveniently selected institutions in Bangladesh. Third- to fifth-year students were invited to participate through a self-administered questionnaire. Statistical analyses were performed with the chi-square test and logistic regression to investigate the associated factors.

**Results:**

Overall, 62.4 % students reported exposure to occupational hazards. Physical hazards were the most frequently reported (44.2 %), followed by chemical (19.4 %), psychosocial (17.6 %), biological (13.6 %), and ergonomic hazards (9.1 %). Senior-level students reported a higher exposure to hazards than 3rd year students (4th year: adjusted odds ratio [AOR] = 2.45, 95 % confidence interval [CI] = 1.04, 5.74; 5th year: AOR = 2.43, 95 % CI = 1.30, 4.55). Those who received formal training outside the curriculum were more likely to report exposure (AOR = 2.49, 95 % CI = 1.16, 5.36). Moreover, a higher knowledge level was a protective factor for occupational hazards (AOR = 0.56, 95 % CI = 0.31, 0.99).

**Conclusion:**

Our findings emphasize the need for training programs both within and beyond the curriculum to improve students' practical skills, particularly among senior-level students involved in clinical duties.

## Highlights

• Occupational hazard exposure was reported by 62.4 % of the veterinary students.
• Physical hazard was the most prevalent.
• Senior-level students had higher exposure to hazards.
• Formal training outside the curriculum increased the exposure report.
• Higher knowledge level acted as a protective factor.

## 1 Introduction

Occupational hazards refer to any harm, including injury, illness, or death, arising from the tasks associated with the workplace environment (WHO, 2025). The burden of occupational hazards can cause severe economic losses, accounting for a loss of 5.8 % of the global gross domestic product in 2019, which is significantly higher than the previous estimate of a 4 % loss in 2003 (ILO, 2003; Takala et al., 2024). Beyond the economic consequences, occupational hazards can reduce the quality of life or even cause fatalities (Wong and Chan, 2018). According to the International Labour Organization (ILO), approximately 3 million workers lose their lives every year due to workplace hazards (ILO, 2023). Due to the nature of their profession, veterinarians are also at risk of exposure to occupational hazards, highlighting significant public health risks (Mishra and Palkhade, 2020).

Veterinarians work in a wide range of environments, including clinics, laboratories, farms, zoos, and educational institutions (U.S. Bureau of Labor Statistics, 2025), where they are exposed to various hazards. These can include physical, chemical, biological, ergonomic, and psychosocial hazards (Che Huei et al., 2020; Wilburn and Eijkemans, 2004). Physical injuries are a common concern among veterinarians, with frequent cases involving sharp instrument accidents, musculoskeletal strains, and animal-related incidents, such as bites, scratches, and kicks (Epp and Waldner, 2012). Besides physical injuries, long-term exposure to radiation, such as X-rays can lead to reproductive problems, birth defects, and even cancer (CDC, 2023; Linet et al., 2010). Chemical agents, such as anesthetics, pesticides, and cytotoxic drugs can also lead to similar adverse effects (Epp and Waldner, 2012; Olika et al., 2022; Shirangi et al., 2009).

Zoonotic diseases are one of the primary concerns among veterinarians, as they are constantly in contact with diseased animals (Kinnunen et al., 2022). The prevalence and risk of these diseases can vary across countries. For instance, a study among veterinarians in the USA reported an 8 % prevalence of zoonotic diseases such as campylobacteriosis, salmonellosis, brucellosis, and Q-fever (Cherry et al., 2022). However, higher rates were reported among veterinarians in Finland (15 %) and the UK (24.6 %), approximately two to three times greater than that observed in the USA (Kinnunen et al., 2022; Robin et al., 2017). Another study in Finland found viral, bacterial, and parasitic zoonoses among veterinarians, where the participants reported being exposed to potentially fatal organisms, such as *Bacillus anthracis* and the Rabies virus (Jokelainen et al., 2024). In Bangladesh, anthrax, avian influenza virus, and Nipah virus infections are some of the common and recurrent zoonotic concerns (Chowdhury et al., 2021). This is not only concerning for veterinary professionals, but also for the general population. Veterinarians can act as a carrier for zoonotic diseases to the general population (Baker and Gray, 2009). This is notable since 60 % of all infectious diseases and 75 % of all emerging and reemerging diseases are attributed to zoonotic diseases (CDC, 2024).

Psychosocial risks, often overlooked by physical or biological hazards, are a critical issue among veterinary professionals (Volk, 2022). A survey among Canadian veterinarians observed higher rates of depression, anxiety, burnout, and compassion fatigue compared to the general population (Perret et al., 2020). Additionally, veterinarians are 2.7 times more likely to attempt suicide than the general population (Volk, 2022). Professional mistakes, lack of professional support, work-related stress, including long working hours, conflictual relationships with colleagues and clients, emotional exhaustion, etc., are among the various reasons for such outcomes (Stoewen, 2015). Furthermore, veterinarians face various ergonomic challenges leading to musculoskeletal discomfort (Seagren et al., 2022). Among veterinarians in New Zealand, the most likely tasks leading to musculoskeletal discomfort are lifting, surgery, rectal palpations, and animal handling due to awkward posture, repetitive activities, and physical activities (Scuffham et al., 2010). In Queensland, veterinarians report the most prevalent body sites for musculoskeletal disorders to be the lower back, neck, shoulders, upper back, and knees (Smith et al., 2009).

Veterinary students are exposed to such risks while handling animals during their clinical duties (Adebowale et al., 2020). They are at a particularly greater risk since they have yet to complete their academic training and are not sufficiently trained to safely handle these hazards (Furtado et al., 2024; Johnson and Fritschi, 2024). This is evident by the 31.5 % prevalence of zoonotic disease infections among veterinary students in the UK, which is higher than the 24.6 % reported among British veterinarians (Furtado et al., 2024; Robin et al., 2017). Although occupational hazards are covered and discussed in some institutions during the course of the study, whether the students retain that information or how seriously they perceive it may determine whether or not they take the necessary precautions to protect themselves (Nyokabi et al., 2024). In a developing country like Bangladesh, which lacks resources and facilities, maintaining proper safety practices is more challenging (Akhter et al., 2019), which leaves veterinary students more vulnerable to such hazards.

An investigation into the prevalence and associated factors regarding occupational hazards among veterinary students is necessary to understand the extent of the problem and implement preventive measures. Despite being such a vulnerable group, no study in Bangladesh has explored the occupational hazard risk among veterinary students. At a global level, only a handful of studies have addressed this issue among veterinary students, and these did not encompass the full spectrum of occupational risks (Adebowale et al., 2020; Furtado et al., 2024; Johnson and Fritschi, 2024). This study aims to fill this gap and add to the sparse global literature by comprehensively analyzing the prevalence and factors associated with occupational hazard exposure, which will aid authorities in developing evidence-based policies.

## 2 Methods

### 2.1 Study design and population

A cross-sectional study was conducted between August 25, 2024 and September 10, 2024 among students enrolled in a veterinary medicine program (DVM or B.Sc. Vet. Sci. & A.H.) in Bangladesh. Two academic institutions were selected conveniently for data collection, coded in this study as Vet School 1 (VS1) and Vet School 2 (VS2). Subsequently, all veterinary students in the 3rd, 4th, and 5th year of their undergraduate program were approached to participate in the survey, since they are more vulnerable to occupational hazards due to their frequent contact with animals during clinical duties.

The inclusion criteria for the study were veterinary students in their 3rd, 4th, and 5th academic year who were present in the classroom during data collection and consented to participate. Those who did not meet these inclusion criteria were excluded from the survey.

### 2.2 Data collection tool, pilot study, and reliability testing

A structured self-administered questionnaire was constructed, with mainly closed-ended questions following an extensive literature review. The questionnaire comprised several sections, including socio-demographic and other information (institution, age, sex, religion, source of financial support, accommodation, current year of study, formal training outside the curriculum, and curricular education), familiarity with occupational hazards, knowledge about occupational hazards, attitude towards occupational hazards, and occupational hazards exposure. The questionnaire was administered in English, as the medium of instruction for the veterinary programs at both institutions is English. The detailed questionnaire is provided in Appendix A.

To assess the clarity and timing of the questionnaire, a pilot study was conducted among five participants. Based on the feedback from the students, the questionnaire was modified to improve comprehensibility. Participants from the pilot study were excluded from the final analysis. Cronbach's alpha was calculated to test the internal consistency of the scales used. Cronbach's alpha for the knowledge and attitude scale was 0.70 and 0.84, respectively, indicating good internal consistency.

### 2.3 Sample size calculation

The required sample size was calculated using Cochran's formula, assuming a 50 % proportion (p), 95 % confidence interval (Z = 1.96), and a 5 % margin of error (e) due to the absence of a similar study from Bangladesh. This yielded an uncorrected sample size of 384. Given the estimated population size of approximately 2100 veterinary students in the 3rd to 5th years nationwide, the corrected sample size was calculated using the finite population correction formula, resulting in a sample size of 325. To ensure adequacy, the final sample size was rounded up to 330. The detailed calculation is provided in Appendix B.

### 2.4 Measures

#### 2.4.1 Socio-demographic and other information

Information regarding socio-demographic characteristics was collected in this study. Data regarding age were collected in a continuous format and subsequently categorized into ≤22 years, 23–24 years, and ≥ 25 years. Moreover, students following Buddhism and Christianity were merged to form the ‘Others’ group in the religion variable. Training outside the curriculum indicated participation in any structured seminars, trainings, or workshops on occupational safety and health (OSH) beyond their degree program or curriculum.

#### 2.4.2 Familiarity with occupational hazards

Familiarity with occupational hazards was evaluated using a binary-response (Yes/No) single-item question: ‘Do you know what an occupational hazard is?.’ Those who responded ‘Yes’ were categorized as being familiar with occupational hazards and were asked to complete the subsequent knowledge-related items. Those who responded ‘No’ were considered unfamiliar with occupational hazards and thus, were instructed to skip the knowledge-related section and proceed with the rest of the survey.

#### 2.4.3 Knowledge about occupational hazards

To assess the students' knowledge, respondents who were familiar with occupational hazards were given 19 questions to respond to, some of which were multiple-choice questions, while others were multiple-answer questions. Each correct and incorrect response was marked with 1 and 0 points, respectively, while partially correct answers (only for multiple-answer questions) were marked with 0.5 points. Subsequently, the points for all 19 items were summed to obtain the total knowledge score for an individual, ranging from 0 to 19. The median score was used to categorize the knowledge and attitude scales to ensure adequate statistical power and balanced group sizes for meaningful comparison. This approach has been used in previous studies exploring knowledge, attitude, and practice levels to provide sample-specific categorization (Aluko et al., 2016; Muhammad et al., 2021). For the knowledge scale, a median score of 14.5 was used as a cut-off.

#### 2.4.4 Attitudes towards occupational hazards

Attitudes of the participants were evaluated using eight statements, with responses on a 5-point Likert scale (1 = strongly disagree, 2 = disagree, 3 = neutral, 4 = agree, and 5 = strongly agree). The total score ranged from 8 to 40, and the cut-off for dichotomizing into higher and lower levels of attitudes was based on the median score (32).

#### 2.4.5 Occupational hazard exposure

To determine whether the students ever faced any occupational hazards in the academic setting, a 1-item question was used: ‘Have you ever experienced any occupational hazard incidents in your educational setting’, with a binary response of ‘Yes’ or ‘No’. Additionally, the participants were asked about the types of occupational hazards they had been exposed to, with response options including physical, chemical, biological, ergonomic, and psychosocial hazards.

### 2.5 Statistical analysis

Descriptive and inferential statistics to explore the prevalence and associated factors regarding occupational hazards were performed. Categorical variables were reported as frequencies and percentages, whereas mean and standard deviation were presented for the continuous variables. Pearson's chi-square test was utilized to investigate the association between the study variables and occupational hazard exposure, since both the predictor variables and the binary outcome were categorical. Multivariable logistic regression with backward elimination technique was used to identify the factors associated with the outcome variables, allowing for the adjustment of potential confounding factors. The results are presented as adjusted ORs (AOR) with 95 % confidence intervals (CI). Age was excluded from the regression model to avoid collinearity issues with the current year of study. A significance level of *p* < 0.05 with a 95 % confidence interval was adopted for all statistical analyses. IBM SPSS version 25 for Windows (IBM Corp., Armonk, NY, USA) was used for statistical analyses, while 95 % confidence intervals for prevalence estimates were calculated using Stata version 17 (StataCorp, College Station, TX, USA). Microsoft Excel 2016 (Microsoft Corp., Redmond, WA, USA) was used for graphical presentation.

### 2.6 Ethical approval

The Ethical Committee of Chattogram Veterinary and Animal Sciences University (CVASU) approved this research on August 19, 2024 (Ref: CVASU/Dir(R&E)EC/2024/763/7). The study adhered to institutional guidelines for the protection of human subjects, encompassing safety and privacy considerations. The study participants were briefed about the objectives, procedure, potential risks, and benefits of the study. Subsequently, written consent was obtained prior to the survey, which reiterated the study's objectives, benefits, estimated duration, participants' right to withdraw at any time without negative consequences, and the assurance of complete confidentiality. Moreover, no compensation was offered to the participants to avoid coercion.

## 3 Results

### 3.1 Characteristics of the participants and associations with occupational hazard exposure

Out of a total of 330 veterinary students, 43.0 % were from Vet School 1 (VS1), and 57.0 % were from Vet School 2 (VS2). The majority of students were aged 23–24 years (57.7 %), male (64.8 %), Muslim (79.9 %), and dependent on family for their financial source (60.6 %). Notably, 37.0 %, 17.3 %, and 45.8 % of the students were in the 3rd, 4th, and 5th year of their study program, respectively. The majority of them lived on-campus (81.7 %). Over half of the respondents received OSH education from their curriculum (57.0 %), while very few of the respondents took any formal OSH training outside their curriculum (18.2 %). Notably, 77.9 % of the students were aware of the term ‘occupational hazard’ before the survey, and less than half of them had a higher level of knowledge regarding the subject matter. Similarly, only 45.8 % had higher attitudes regarding occupational hazards (Table 1).

**Table 1:** Descriptive statistics of the predictor variables among veterinary students in Bangladesh, 2024.

Variables	N (%)
Institution	
VS1	142 (43.0)
VS2	188 (57.0)
Age group	
≤22 years	65 (20.4)
23–24 years	184 (57.7)
≥25 years	70 (21.9)
Sex	
Male	214 (64.8)
Female	116 (35.2)
Religion	
Islam	263 (79.9)
Hinduism	53 (16.1)
Others	13 (4.0)
Source of financial support	
Family	200 (60.6)
Self-earning	44 (13.3)
Mixed	86 (26.1)
Accommodation	
On-campus	268 (81.7)
Off-campus	60 (18.3)
Current year of study	
3rd year	122 (37.0)
4th year	57 (17.3)
5th year	151 (45.8)
Formal training outside the curriculum	
No	270 (81.8)
Yes	60 (18.2)
Curricular education on occupational safety and health	
No	142 (43.0)
Yes	188 (57.0)
Familiarity with occupational hazards	
No	73 (22.1)
Yes	257 (77.9)
Knowledge about occupational hazards	
Lower	138 (53.7)
Higher	119 (46.3)
Attitudes towards occupational hazards	
Lower	179 (54.2)
Higher	151 (45.8)

The chi-square test revealed several associations between the study variables and exposure to occupational hazards. Students' year of study was significantly associated with the outcome (*p* = 0.01), where 4th year students had a higher prevalence of exposure compared to 5th year and 3rd year students. Participants who received any kind of training related to OSH outside their curriculum were more likely to be exposed to occupational hazards than those who did not (*p* < 0.01). Following a similar pattern, having OSH education incorporated into their curriculum was associated with a higher risk of occupational hazards (*p* = 0.02; Table 2).

**Table 2:** Association between the predictor variables and occupational hazard exposure among veterinary students in Bangladesh, 2024.

Variables	Occupational hazard exposure		*p*-value⁎
	No, N (%)	Yes, N (%)	
Institution			
VS1	53 (37.3)	89 (62.7)	0.94
VS2	71 (37.8)	117 (62.2)	
Age group			
≤22 years	26 (40.0)	39 (60.0)	0.79
23–24 years	69 (37.5)	115 (62.5)	
≥25 years	24 (34.3)	46 (65.7)	
Sex			
Male	83 (38.8)	131 (61.2)	0.54
Female	41 (35.3)	75 (64.7)	
Religion			
Islam	92 (35.0)	171 (65.0)	0.12
Hinduism	25 (47.2)	28 (52.8)	
Others	7 (53.8)	6 (46.2)	
Source of financial support			
Family	68 (34.0)	132 (66.0)	0.13
Self-earning	16 (36.4)	28 (63.6)	
Mixed	40 (46.5)	46 (53.5)	
Accommodation			
On-campus	97 (36.2)	171 (63.8)	0.30
Off-campus	26 (43.3)	34 (56.7)	
Current year of study			
3rd year	59 (48.4)	63 (51.6)	0.01
4th year	16 (28.1)	41 (71.9)	
5th year	49 (32.5)	102 (67.5)	
Formal training outside the curriculum			
No	114 (42.2)	156 (57.8)	<0.01
Yes	10 (16.7)	50 (83.3)	
Curricular education on occupational safety and health			
No	64 (45.1)	78 (54.9)	0.02
Yes	60 (31.9)	128 (68.1)	
Familiarity with occupational hazards			
No	32 (43.8)	41 (56.2)	0.21
Yes	92 (35.8)	165 (64.2)	
Knowledge about occupational hazards			
Lower	44 (31.9)	94 (68.1)	0.16
Higher	48 (40.3)	71 (59.7)	
Attitudes towards occupational hazards			
Lower	60 (33.5)	119 (66.5)	0.10
Higher	64 (42.4)	87 (57.6)	

⁎ *p*-values were obtained using the chi-square test.

### 3.2 Prevalence of occupational hazards

**Fig. 1:**
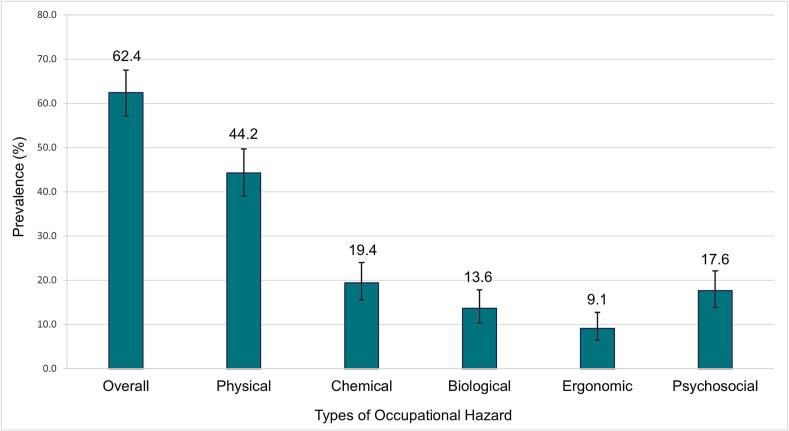
Prevalence of occupational hazards among veterinary students in Bangladesh, 2024. The error bars represent 95 % confidence intervals.

Overall, 62.4 % (95 % CI = 57.1 %, 67.5 %) of students reported facing at least one type of occupational hazard in their academic or clinical settings. The most common types of occupational hazards reported by the students were physical hazards (44.2 %, 95 % CI = 39.0 %, 49.7 %), followed by chemical hazards (19.4 %, 95 % CI = 15.5 %, 24.0 %), psychosocial hazards (17.6 %, 95 % CI = 13.8 %, 22.1 %), biological hazards (13.6 %, 95 % CI = 10.3 %, 17.8 %), and ergonomic hazards (9.1 %, 95 % CI = 6.4 %, 12.7 %; Fig. 1).

### 3.3 Multivariable logistic regression analysis to evaluate the strength of association of predictors with occupational hazards exposure

The final adjusted binary logistic regression model found three associated factors for occupational hazard exposure. When compared to the 3rd year students, 4th year (AOR = 2.45, 95 % CI = 1.04, 5.74) and 5th year (AOR = 2.43, 95 % CI = 1.30, 4.55) students were more likely to experience occupational hazards, respectively. Those who participated in training programs for occupational hazards were more likely to report exposure compared to those who did not (AOR = 2.49, 95 % CI = 1.16, 5.36). Moreover, having good knowledge regarding occupational hazards was found as a protective factor for their encounters (AOR = 0.56, 95 % CI = 0.31, 0.99; Table 3).

**Table 3:** Multivariable logistic regression analysis of factors associated with occupational hazard exposure among veterinary students in Bangladesh, 2024.

Variables	Adjusted Odds Ratio⁎ (95 % Confidence Interval)
Current year of study	
3rd year	1.00
4th year	2.45 (1.04, 5.74)
5th year	2.43 (1.30, 4.55)
Formal training outside the curriculum	
No	1.00
Yes	2.49 (1.16, 5.36)
Knowledge about occupational hazards	
Lower	1.00
Higher	0.56 (0.31, 0.99)

⁎ The adjusted odds ratios were adjusted for current year of study, formal training outside the curriculum, and knowledge about occupational hazards.

## 4 Discussion

This study provides a comprehensive overview of occupational hazards exposure among veterinary students in Bangladesh. The study revealed that 62.4 % of the students encountered occupational hazards in their academic or clinical settings. The study year, formal training outside the curriculum, and knowledge levels were the determining factors for occupational hazards exposure.

The present study found that 44.2 %, 19.4 %, and 13.6 % of the veterinary students reported experiencing physical, chemical, and biological hazards, respectively. A similar proportional pattern was observed in a systematic review and meta-analysis regarding veterinary occupational hazards, where 65.0 %, 7.0 %, and 17.0 % prevalence was reported for physical, chemical, and biological hazards, respectively (Adebowale et al., 2021). Likewise, a study among Indian veterinarians found that more than half (54.7 %) of the participants suffered from work-related injuries, with large-animal veterinarians being twice as likely to be at risk (Mishra and Palkhade, 2020). Furthermore, a survey conducted on Nigerian veterinary students reported exposure to various physical hazards, such as animal scratches, animal kicks, falls/slips, and animal bites (Adebowale et al., 2020). This indicates that physical hazards are a consistently common concern in the veterinary field across various educational and occupational settings, regardless of the country. However, the exposure to chemical hazards was much greater among veterinary students in the current study than among professionals (Adebowale et al., 2021), which might be attributed to their frequent involvement in laboratory-based practical sessions where chemicals are commonly used. This is evident among Nigerian veterinary clinical students, where 27.8 % and 29.0 % of the participants experienced eye burns and choking incidents, which were associated with exposure to formalin during anatomy practical sessions (Adebowale et al., 2020). Additionally, the prevalence of biological hazard exposure among veterinary students in this study was slightly lower than in practicing veterinarians (Adebowale et al., 2021). This finding is expected since veterinarians are more frequently involved in clinical work and animal handling. However, the prevalence of such hazards among veterinarians was found to vary depending on their country of origin, such as 8 % in the USA (Cherry et al., 2022), 15 % in Finland (Kinnunen et al., 2022), 24.6 % in the UK (Robin et al., 2017), and 49.3 % in India (Palkhade et al., 2022). Regardless, such high prevalence is concerning since the majority of the veterinary students in Bangladesh are not vaccinated against common zoonotic threats (Sayed et al., 2022), whereas such vaccination is a requirement prior to enrollment in US universities (Lindenmayer et al., 2013).

Ergonomic and psychosocial hazards, although often overlooked, are a significant concern among veterinary students. In this study, 9.1 % of the veterinary students suffered from ergonomic hazards associated with their academic and clinical environments. This is reflected in a study among veterinary medicine students, which showed an early onset of musculoskeletal discomfort, particularly in the neck and lower back regions (Grünwald and Licka, 2023). The study also highlighted the importance of microbreaks in pain reduction and improving self-efficacy in handling physically demanding tasks. Similarly, a survey among Canadian bovine veterinarians found that 15.5 % of the participants experienced upper extremity musculoskeletal discomfort in the preceding year (Reist et al., 2020), which is higher than the rate of ergonomic hazard exposure found in this study. This might be because large animal practice requires more physically demanding tasks, leading to higher ergonomic risks (Thielmann et al., 2024). Furthermore, 17.6 % of the students in this study reported exposure to psychosocial risks, aligning with the 22.6 % prevalence of depression found among US veterinary students (Nahar et al., 2019). Key stressors affecting veterinary students may include academic workload, frequent assessment, challenges in program implementation, financial burden, and coping difficulties. In contrast, healthy personal habits, social support, and organizational strength might act as protective factors for the mental well-being of veterinary students (Weston et al., 2017).

The final adjusted logistic regression model identified academic year, formal training outside the curriculum, and knowledge level as significant predictors of occupational hazard exposure. The prevalence of exposure to occupational hazards was reported to be 2.45 times and 2.43 times higher among 4th and 5th year students, respectively, compared to the 3rd year students. This resonates with a study among medical students in Germany, where students in their higher academic year reported higher needle-stick injuries, with prevalence ranging from 12 % in 1st year students to 41 % in 4th year students (Deisenhammer et al., 2006). This observation may be explained by senior veterinary students having increased, more advanced, and frequent clinical responsibilities than their junior counterparts. Moreover, higher academic level students exhibited a higher level of knowledge (23.5 % vs. 46.2 % vs. 60.9 % for 3rd, 4th, and 5th year, respectively), which might be attributed to higher reporting of the incidents.

Notably, students with training outside the curriculum reported higher levels of exposure to occupational hazards compared to those without. Trained personnel are expected to be more aware of the hazards in their workplace (Brunette, 2005). Thus, they might report incidents of hazard exposure more frequently than those who are not trained. Moreover, poor occupational safety training can also be linked with higher rates of exposure to hazards (Adebowale et al., 2020). Contrary to our findings, a prospective study involving French apprentices and students entering the workforce reported that those who received OSH education at school had half the rate of workplace injuries (Boini et al., 2017). However, the study focused on curricular education rather than extracurricular training. A similar observation was reported in an intervention study among Chinese nursing students (Yao et al., 2013). Future research should explore these discrepancies by assessing the effectiveness of curricular OSH education compared to supplemental training programs.

The odds of encountering occupational hazards were approximately half among the students with higher knowledge levels compared to their counterparts with lower knowledge levels. This is consistent with the findings among Taiwanese dentists, where insufficient knowledge was associated with a higher exposure to occupational needle-stick injuries (Cheng et al., 2012). Similar findings are also evident across other occupational groups (Thomas et al., 2023). This finding is expected since the OSH knowledge enables students or professionals to identify occupational hazards and adopt safer practices (Nyokabi et al., 2024).

This is the first study to evaluate the prevalence and associated factors of occupational hazards among veterinary students in Bangladesh, addressing a critical gap in the existing literature, particularly in the context of Bangladesh. By identifying key risk factors and types of hazards, the findings can inform future safety protocols, educational interventions, and policy decisions targeted specifically towards veterinary students. However, despite its strengths, the study has some limitations. Its cross-sectional design precludes causal inference between the factors and the outcome. Moreover, self-reported data might introduce recall bias. The non-probability sampling method can also lead to selection bias, potentially limiting the generalizability of the findings. Future research should employ a nationwide survey with a probability sampling method to prevent these limitations. A longitudinal intervention study design could also be conducted to assess the effectiveness of various training programs over time, particularly their impact during students' academic stages and subsequently at the professional level.

## 5 Conclusion

In conclusion, this study contributes new evidence to the limited literature by investigating the prevalence and associated factors of occupational hazards among veterinary students in Bangladesh using traditional statistical analysis. The findings suggest that a significant proportion of the students encountered occupational hazards during their academic life, with physical hazards being the most prevalent type. Academic level, formal training outside the curriculum, and knowledge levels were identified as factors associated with occupational hazard exposure. These findings underscore the need for targeted interventions, including safety training and awareness programs both within and beyond the curriculum, to prevent occupational hazard exposure by enhancing students' knowledge, particularly among senior-level students.

## CRediT authorship contribution statement

**Pronab Das:** Writing – review & editing, Writing – original draft, Visualization, Software, Methodology, Investigation, Formal analysis, Data curation, Conceptualization. **Seemanta Das:** Writing – review & editing, Writing – original draft, Conceptualization. **Muhammad Abdul Mannan:** Writing – review & editing, Supervision, Conceptualization. **Sharmin Chowdhury:** Writing – review & editing, Supervision, Project administration, Methodology, Conceptualization.

## Funding information

This research did not receive any specific grant from funding agencies in the public, commercial, or not-for-profit sectors.

## Declaration of competing interest

The authors declare that they have no known competing financial interests or personal relationships that could have appeared to influence the work reported in this paper.

## Data Availability

Data will be made available on request.

## Appendix A Supplementary data

Supplementary material: Questionnaire Image 1

Supplementary material: Sample size calculation Image 2
