# Supplementary material for: Prevalence and factors associated with occupational hazard exposure among undergraduate veterinary students in Bangladesh
Source: Prev Med Rep. 2025 Aug 7;57:103196. doi: 10.1016/j.pmedr.2025.103196 (PMC12359168; doi:10.1016/j.pmedr.2025.103196)
Supplement: Supplementary file 1 — Supplementary material: Questionnaire [file mmc1.docx]

# Appendix A: Questionnaire

**Table 1.** Questionnaire on occupational hazard exposure among veterinary students in Bangladesh (2024)

| **Sl. No.** | **Question** | **Response** |
| --- | --- | --- |
| **Socio-demographic and other factors** | | |
| 1 | Institution Name |  |
| 2 | Age (years) |  |
| 3 | Gender | Male  Female  Others______ |
| 4 | Religion | Islam  Hinduism  Christianity  Buddhism  Others_______ |
| 5 | Source of Financial Support | Family  Self-earning  Mixed |
| 6 | Accommodation | On-campus  Off-campus |
| 7 | Current Year of Study (eg. 3^rd^, 4^th^, 5^th^) | 3^rd^  4^th^  5^th^ |
| 8 | Have you received any formal training or education on occupational hazards outside the curriculum? | Yes  No |
| 9 | Does your curriculum incorporate education on occupational hazards? | Yes  No |
| **Familiarity with Occupational Hazards** | | |
| F1 | Do you know what occupational hazard is? | Yes  No |
| ***If no, skip to Attitudes section*** | | |
| **Knowledge about Occupational Hazards** | | |
| K1 | If yes, which of the statement is correct about occupational hazards? | Workplace incidents that always result in injury  Workplace incidents that always cause diseases  Any potential risk or danger in the workplace that can cause harm  Not sure |
| K2 | Are you aware of the potential occupational hazards in your field of study? | Yes  No |
| K3 | Which of these are occupational hazards? | Physical  Chemical  ☐ Biological  ☐ Ergonomic  ☐ Psychosocial  All of the above  Not sure |
| K4 | What type of occupational hazard is bacteria? | Physical  Chemical  Biological  Ergonomic  Psychosocial  Not sure |
| K5 | What type of occupational hazard is radiation? | Physical  Chemical  Biological  Ergonomic  Psychosocial  Not sure |
| K6 | What type of occupational hazard is cleaning and disinfection agents? | Physical  Chemical  Biological  Ergonomic  Psychosocial  Not sure |
| K7 | What type of occupational hazard is workload demand? | Physical  Chemical  Biological  Ergonomic  Psychosocial  Not sure |
| K8 | What type of occupational hazard is poor posture? | Physical  Chemical  Biological  Ergonomic  Psychosocial  Not sure |
| K9 | During which of the following activities is a needle stick injury most likely to occur? | Administering injection  Recapping needle  Suturing  Disposal of needle |
| K10 | What are the appropriate steps to take in the event of a bite from an animal? | Wash the wound with soap and water  Seek medical attention immediately  Report to the appropriate authorities  All of the above  Not sure |
| K11 | Are you familiar with the potential long-term health effects of chronic exposure to certain occupational hazards, such as radiation, chemicals, or repetitive strain injuries? | Yes  No |
| K12 | Which of these diseases can be spread from animal to human? | Rabies  Anthrax  ☐ Brucellosis  ☐ Ringworm  ☐ Leptospirosis  ☐ Tuberculosis  ☐ Toxoplasmosis  ☐ All of the above  Not sure |
| K13 | Which of these diseases are mainly spread via animal bite or scratch? | Rabies  Anthrax  ☐ Brucellosis  ☐ Ringworm  ☐ Leptospirosis  ☐ Tuberculosis  ☐ Toxoplasmosis  ☐ All of the above  Not sure |
| K14 | Which of these diseases are mainly spread via aerosol/air droplets? | Rabies  Anthrax  ☐ Brucellosis  ☐ Ringworm  ☐ Leptospirosis  ☐ Tuberculosis  ☐ Toxoplasmosis  ☐ All of the above  Not sure |
| K15 | Which of these diseases are mainly spread via direct contact with animals or contaminated materials (eg. water, soil, food, etc.)? | Rabies  Anthrax  ☐ Brucellosis  ☐ Ringworm  ☐ Leptospirosis  ☐ Tuberculosis  ☐ Toxoplasmosis  ☐ All of the above  Not sure |
| K16 | Do you know the appropriate safety protocols to prevent occupational hazards in your field? | Yes  No |
| K17 | If yes, which of these are appropriate safety protocols to prevent occupational hazards? | Risk assessment  Personal Protective Equipment (PPE)  Incident reporting and investigation  All of the above  Not sure |
| K18 | Are you familiar with the use of personal protective equipment (PPE) for occupational safety? | Yes  No |
| K19 | If yes, which of these are personal protective equipment (PPE)? | Gloves  Head Cap  Gumboot  Mask  Goggles  Lab Coat  All  Not sure |
| **Attitude towards Occupational Hazards** | | |
| A1 | I am concerned about occupational hazards in the field settings. | Strongly Disagree  Disagree  Neutral  Agree  Strongly Agree |
| A2 | Occupational hazards can have long-term health effects. | Strongly Disagree  Disagree  Neutral  Agree  Strongly Agree |
| A3 | Adhering to safety practices can significantly reduce the risk of occupational hazards. | Strongly Disagree  Disagree  Neutral  Agree  Strongly Agree |
| A4 | I am comfortable reporting unsafe working conditions or incidents in my educational setting. | Strongly Disagree  Disagree  Neutral  Agree  Strongly Agree |
| A5 | The curriculum should incorporate more emphasis on occupational safety. | Strongly Disagree  Disagree  Neutral  Agree  Strongly Agree |
| A6 | There is a need for more safety awareness campaigns in your field of study. | Strongly Disagree  Disagree  Neutral  Agree  Strongly Agree |
| A7 | There should be better support systems in place for students facing occupational hazards. | Strongly Disagree  Disagree  Neutral  Agree  Strongly Agree |
| A8 | Prevention of occupational hazards is a joint responsibility of the administrator and the students. | Strongly Disagree  Disagree  Neutral  Agree  Strongly Agree |
| **Practices regarding Occupational Hazards** | | |
| P1 | Do you regularly use personal protective equipment (PPE) during practical sessions or clinical work? | Never  Rarely  Sometimes  Often  Always |
| P2 | Which of these types of PPE do you use regularly? | Gloves  Mask  Goggles  Lab Coat  All of them  None |
| P3 | Do you properly dispose of hazardous materials? | Yes  No |
| P4 | Do you recap needles after use? | Yes  No |
| P5 | Are you vaccinated against diseases that may be encountered in your field of study?  (e.g., Rabies) | Yes  No |
| P6 | For which of these diseases are you vaccinated against? | Rabies  Hepatitis  Tetanus  None |
| P7 | Do you practice correct body positioning during clinical practices? | Yes  No |
| **Occupational Hazard Exposure** | | |
| 1 | Have you ever experienced any occupational hazard incidents in your educational setting? | Yes  No |
| 2 | If yes, what type of occupational hazards have you experienced? | Physical  Chemical  ☐ Biological  ☐ Ergonomic  ☐ Psychosocial  All of the above |
