# Supplementary material for: Prevalence and factors associated with occupational hazard exposure among undergraduate veterinary students in Bangladesh
Source: Prev Med Rep. 2025 Aug 7;57:103196. doi: 10.1016/j.pmedr.2025.103196 (PMC12359168; doi:10.1016/j.pmedr.2025.103196)
Supplement: Supplementary file 2 — Supplementary material: Sample size calculation [file mmc2.docx]

**Sample size calculation**

Cochran's formula was used to estimate the sample size required for the study (Cochran, 1977). Since there was no similar study from Bangladesh, the following assumptions were made: 50% proportion (p), 95% confidence interval (Z = 1.96), and a 5% margin of error (e).

$n_{0}= \frac{Z^{2}p (1-p)}{e^{2}}$

$n_{0}= \frac{{(1.96)}^{2} \times0.5 (1-0.5)}{{0.05}^{2}}$ = 384

However, since the estimated population size for the study was approximately 2,100 (total number of 3^rd^, 4^th^, and 5^th^ year veterinary students in Bangladesh), which was less than 10,000, we used the following correction formula (Thrusfield, 1995):

$n= \frac{n_{0}}{(1+{n_{0}}/{N)}}$

$n= \frac{384}{(1+{384}/{2100)}}$ = 325.42

Where,

n = corrected sample size,

$n_{0}$ = uncorrected sample size, and

N = finite population size.

However, to ensure adequacy, the final sample size was rounded up to 330.

**References**

Cochran, W. G. (1977). *Sampling techniques* (3rd ed.). John Wiley & Sons Inc. https://www.wiley.com/en-us/Sampling+Techniques%2C+3rd+Edition-p-9780471162407

Thrusfield, M. (1995). *Veterinary Epidemiology* (2nd ed.). Blackwell Science Ltd.
